# Second Impregnation at the Fourth Month of Utero-Gestation

**Published:** 1859-04

**Authors:** James Paerson Irvine

**Affiliations:** Surgeon to the Union of Lancaster. Galgate, by Lancaster


*Second Impregnation at the Fourth Month of Utero-Gestation*

Sir,—Mrs. S., aged 22, a stout, healthy looking young woman, commenced labor of her first child on the evening of November 7, the pains continuing at irregular intervals till the 11th, in the afternoon of which day I was called in, and found the pains, though short and ineffectual, occurring at regular intervals of five minutes. Upon inquiry, I ascertained that she had been married two years, and that eighteen months ago she had an abortion at the tenth week, from the effects of which she quickly recovered, regaining her usual strength in a suprising manner till the commencement of her present conception, since which time she has been in a moderately healthy condition. About four or five months ago she was, according to her own statement, seized with the impression that she was conceiving twins, and had subsequently at various times made mention of the same to her relatives. Finding, on examination, a rigid os uteri, I left her, informing the nurse to send for me when the pains became stronger. At 11 o'clock the same evening I was again summoned to my patient, and to my utter astonishment, found presenting umbilically, what I supposed to be a premature fœtus, which by a few further efforts of the uterus, was expelled with a gush of liq. amnii. This I concealed under the bed-clothes, informing my patient and the bystanders that it was merely the passage of a few clots of blood. I now laid my hand on the abdomen, and found the cavity of the uterus still occupied; and examining *per vaginam* I discovered the head of a fœtus, pushing before it a dense bag of liq. amnii. The pains gradually increased in strength and efficacy till 2 o'clock, A. M., when the natural delivery of a mature, fullgrown fœtus took place, and shortly after the placenta was expelled. I then directed the all-inquisitive nurse to go down stairs and make the mother a cup of tea, and during her absence I ascertained that the mass first expelled was a fœtus of from four to five months, in a high state of preservation, attached by its cord to a separate placenta, which was intimately blended with that of the mature fœtus; still there was a distinct line of demarcation between the two.

Now the question of superfœtation is one of the unsettled points in the profession, and for that reason I have thought it advisable to bring forward the facts of the above case, testifying that superfœtation may occur even at the fourth month of utero-gestation. Can it possibly be argued, in contradiction to this view, by the supporters of the theory of non-superfœtation, that this fœtus presenting no abnormal peculiarities was arrested in its development at the fourth or fifth month, and yet lived the full period of pregnancy?

I am, etc.,

James Paerson Irvine,
Surgeon to the Union of Lancaster.

Galgate, by Lancaster.

[*New Orleans Med. News & Hos. Gaz.*

